# Construction of *Streptomyces coelicolor* A3(2) mutants that exclusively produce NA4/NA6 intermediates of agarose metabolism through mutation induction

**DOI:** 10.1038/s41598-023-46410-7

**Published:** 2023-11-03

**Authors:** Jina Kim, Eun Joo Kim, Hye-Jeong Ko, Yeon-Hee Lee, Soon-Kwang Hong, Miyoung Shin, Je Hyeon Lee, Woori Kwak

**Affiliations:** 1 Department of Medical and Biological Sciences, The Catholic University of Korea, Bucheon, 14662 Republic of Korea; 2 Dyne Bio Inc., Seongnam-si, Gyeonggido 13209 Republic of Korea; 3 Department of Biological Science and Bioinformatics, Myongji University, 116 Myongji-Ro, Cheoin-gu, Yongin, 17058 Gyeonggido Korea; 4 Department of Pathology, Yale University School of Medicine, New Haven, CT 06510 USA

**Keywords:** Bacteria, Microbiology, Bacterial genes, Bacterial genetics, Molecular evolution

## Abstract

NA4/NA6, an intermediate degradation product of β-agarase, is a high value-added product with anticancer, anti-obesity, and anti-diabetic effects. Therefore, a method that enables the efficient production of NA4/NA6 would be useful from economic and medical perspectives. In this study, we aimed to generate a *Streptomyces coelicolor* A3(2) mutant M22-2C43 that produces NA4/NA6 as a final product; this method serves as a more efficient alternative to the enzymatic conversion of β-agarase for the generation of these products. The M22-2C43 strain was generated through two rounds of mutagenesis and screening for increased β-agarase activity and effective production of NA4/NA6. We assembled the complete genomes of two mutants, M22 and M22-2C43, which were identified following a two-round screening. Large and small genetic changes were found in these two mutants, including the loss of two plasmids present in wild-type *S. coelicolor* A3(2) and chromosome circularization of mutant M22-2C43. These findings suggest that mutant M22-2C43 can produce NA4/NA6 as a degradation product due to functional inactivation of the *dagB* gene through a point mutation (G474A), ultimately preventing further degradation of NA4/NA6 to NA2. To our knowledge, this is the first report of a microbial strain that can effectively produce NA4/NA6 as the main degradation product of β-agarase, opening the door for the use of this species for the large-scale production of this valuable product.

## Introduction

Agar is a polysaccharide composed of agarose and agaropectin, and small amounts of protein, ash, and fat^1,2^. Among them, the unit of agarose called agarobiose, in which D-galactose and 3,6-anhydro-L-galactose are linked through β-1,4 bonds. Agarose has a linear structure, in which agarobiose is repeatedly linked through α-1,3 bonds, and can be degraded by agarase^3–6^. Agarase is divided into α-agarase (EC 3.2.1.158) and β-agarase (EC 3.2.1.81) according to the type of bond they catalyze^7,8^. α-agarase acts on the α-1,3 bond of agarose and produces agarooligosaccharide with 3,6-anhydro-L-galactose as a non-reducing end^9^. On the other hand, β-agarase acts on the β-1,4 bond of agarose and produces neoagarooligosaccharide (NAO) with D-galactose as a non-reducing end^10^. Agarooligosaccharide from α-agarase and NAO from β-agarase have different physiological properties due to structural differences in their non-reducing ends^11^. NAO from β-agarase is classified into neoagarobiose (NA2), neoagarotetraose (NA4), neoagarohexaose (NA6), and neoagarooctaose (NA8) according to the number of linked oligosaccharides. Among these, NA4 and NA6 are known to exhibit medical activity, including anti-obesity^12^, anti-diabetic^13^, improvement of metabolic diseases (such as anti-hyperlipidemic)^14^, anticancer^13^, and immunity-enhancing effects^15,16^. Therefore, efficient production of NA4 and NA6 is important for medical purposes.

*Streptomyces coelicolor* A3(2), a representative strain of *S. coelicolor*, has a linear genome containing two plasmids that can degrade agarose using extracellularly secreted β-agarase^17–19^. Genome analysis of *S. coelicolor* A3(2) revealed that its β-agarases are encoded as *dagA* and *dagB*, which belong to the glycoside hydrolase (GH) family in the enzyme classification system. β-agarase can be classified into various families, such as GH16, GH50, GH86, and GH118, based on the structure and sequence in the CAZy database (http://www.cazy.org/)^20,21^. For *S. coelicolor* A3(2), DagA and DagB β-agarases belong to the GH16 and GH50 families, respectively. DagA and DagB in *S. coelicolor* A3(2) are known to cooperatively degrade agar^22,23^ via the following mechanism: first, agarose is degraded to NA4/NA6 by extracellular DagA (SCO3471), and then NA4/NA6 is degraded into NA2 by extracellular DagB (SCO3487). Finally, decomposed NA2 is degraded to 3,6-anhydro-L-galactose and D-galactose when transported into cells and used as a carbon source^6,11^.

In addition to the enzymatic methods described above, chemical methods can be used for agarose degradation. Enzymatic methods have attracted greater attention than chemical-based methods due to their reliability, high activity, and environmental protective ability^2^. Therefore, various studies have focused on effectively producing an enzyme-like DagA β-agarase, which can produce high value-added NAO^24–27^. To yield NA4/NA6 as a final product, the DagA β-agarase can be obtained by producing a transformed strain or purifying only DagA from the expressed β-agarase^23,28,29^; however, UV-induced mutagenesis was not employed in previous studies.

In this study, M22-2C43, a mutant strain of *S. coelicolor* A3(2), was obtained using UV irradiation. This strain was screened according to the increased β-agarase activity phenotype and was verified to effectively produce NA4/NA6 as a final product. To reveal the genetic background of this phenotype, we assembled the complete genome and conducted comparative genome analysis. To our knowledge, this study is the first to use a mutagenesis strategy to produce high-quality NA4/NA6 without complex processes, such as transformation or DagA purification, and is expected to be the basis for the application of mutagenesis strategies in other species for similar purposes.

## Method and materials

### β-agarase specific activity using the DNS method

The β-agarase DagA activity is measured by a modified 3,5-dinitrosalicylic acid method, as reported to Miller^30^. Briefly, 0.125 mL of the partially purified enzyme solution was mixed with 0.125 mL of 10 mM phosphate buffer (pH 7.0) containing 1% dissolved agar. After the reaction for 10 min at 45 °C, 0.75 mL of dinitrosalicylic acid (DNS) reagent (6.5 g DNS, 2 M NaOH 325 mL, glycerol 45 mL per 1 L distilled water) was added and boiled for 5 min. After cooling, 2 ml of water is added and the optical density at 540 nm (OD540) of the mixture was measured using UV-is spectrophotometer. One unit of enzyme activity is the amount of enzyme that produces reducing sugars equivalent to 1 μmol of glucose per minute from a substrate.

### Mutation induction of *S. coelicolor* A3(2)

For the first round of UV-induced mutagenesis, after the wild-type *S. coelicolor* A3(2) (John Innes Foundation, United Kingdom) was cultured on an actinomycete minimal medium (MM) plate, as reported by Hopwood^31^, for 5 days, 2 mL of 20% (w/v) glycerol was dispensed for spore collection. Wild-type spore stock (1 µL) and TSB (10 mL) were diluted, and culture medium was collected via UV irradiation (30 cm, 30 W, 45 min) in the dark. This culture medium was spread on MM agar plate and cultured at 28 °C for 8 days under dark conditions. The number of surviving colonies on the plate was counted, and 1400 species with large clear zones were screened via staining with Congo red. Colonies were dispensed on a glass filter paper spread with liquid MM (including 2% (w/v) agarose as a carbon source) and cultured at 28 °C for 5 days. A total of 313 species with high spore forming activity were subjected to a secondary screen. The secondary screened species were cultured in RSM3 broth (containing 2% (w/v) agarooligosaccharide) and the culture medium was centrifuged to obtain the supernatant. The supernatant was sterilized and filtered using a syringe filter to obtain the purified supernatant. β-agarase activity in the supernatant of the screened species and wild-type was measured using the DNS method. Compared to the wild-type, the mutant with the highest β-agarase activity was named *S. coelicolor* A3(2) M22.

For a second round of UV-induced mutagenesis*, S. coelicolor* A3(2) M22 spore stock was prepared via culture on an actinomycetes complete medium ISP4 plate as reported by Shirling^32^. Spore stocks of mutant M22 (1 µL) and TSB (5 mL) were diluted, and culture medium was collected via UV irradiation (35–50 cm, 30–40 W, 24–60 min) in the dark. This culture medium was spread on MM agar plate and cultured at 28 °C for 5 days under dark conditions. The surviving colonies on the plate were counted and stained with Lugol’s iodine. After comparing colony sizes, the strains were classified by morphology. The screened strain was inoculated into a liquid medium containing 0.5% (w/v) agarose (including MgCl_2_•7H_2_O 5 g, yeast extract 11 g, and CaCO_3_ 0.5 g based on 1 L of distilled water) and cultured for 2.5 days under 28 °C and 216 rpm conditions. After collecting the supernatant as previously described, the activities of β-agarase and DagA were measured. The mutant with the highest activity was named *S. coelicolor* A3(2) M22-2C43.

### Protein purification

Each strain was inoculated into 1000 mL of liquid medium containing 0.5% (w/v) agarose (including MgCl_2_·7H_2_O 5 g, yeast extract 11 g, and CaCO_3_ 0.5 g based on 1 L of distilled water) and incubated at 28 °C with shaking at 216 rpm for 2.5 days. Thereafter, the culture medium was centrifuged to remove cell debris and the supernatant was collected. Using the collected supernatant as a sample, β-agarase activity was measured and DagA enzyme activity was evaluated. The supernatant was sterilized and filtered using a syringe filter to collect the purified supernatant. Ammonium sulfate was added to the purified supernatant to obtain protein saturation concentrations of 50% and 70%, respectively. The β-agarase enzyme was precipitated via ammonium sulfate precipitation (ASP)^33^, and a pellet-type purified β-agarase enzyme was obtained through centrifugation.

### NAO composition analysis

Agarose was digested by reacting it with a 70% ASP sample obtained through the cultivation of mutant M22-2C43^13,34^. The composition of the NAO product was analyzed using HPLC (Shimadzu Co., Japan) equipped with an LC-20AD Pump. ELSD (Sedere Co., France) was used for the analysis, with ShodexTM AsahipakTM NH2P-50 4E column (4.6 mm ID × 250 mm, 5 μm, Tokyo, Japan). NA4 and NA6 standards were purchased from Qingdao Hehai Biotech Co., Ltd., China. The mobile phase consisted of acetonitrile and water (65:35 v/v). The flow rate was set at 1 mL/min and the column oven temperature was set at 40 °C. The ELSD detector-nebulizer temperature was 50 °C. Under the experimental conditions, the run time was 20 min, and the injection volume was 10 µL. NAO powder was dissolved in acetonitrile and water solution and then the solution was syringe-filtered using a membrane filter (0.22 μm, PVDF, Jet Biofil). The HPLC chromatograms confirmed the identification of NA2, NA4, and NA6, with retention times of 4.69 min, 5.73 min, 7.29 min, 9.55 min, and 12.64 min, respectively.

### Whole genome sequencing

The DNA of two mutants (M22 and M22-2C43) was extracted using the Omega Bio-tek Mag-Bind Universal Pathogen Kit, according to the manufacturer's protocol. Sequencing libraries for Nanopore Flongle and Illumina MiSeq were constructed using the Nanopore SQK-LSK109 and Illumina TruSeq Nano DNA Sample Preparation Kits, respectively. Nanopore base-calling was conducted using Guppy v6.0.6^35^. Quality pass reads from Guppy were assembled using Canu v2.1.1^36^. For the mutant M22-2C43, which has a circular chromosome, the constructed genome sequence was manually trimmed. Hybrid polishing was performed using Nanopore Long and Illumina Short Reads. Medaka v1.6.1 (https://github.com/nanoporetech/medaka) was used with a high-accuracy calling model, and Homopolish v0.3.3^37^ was used for second-level polishing with -m R9.4.pkl parameter. The final polishing process was conducted using Pilon v1.24^38^. For the Pilon polishing process, sequencing artifacts and low-quality bases from the Illumina MiSeq were removed using Trimmomatic v0.39^39^ with ILLUMINACLIP:TruSeq3-PE.fa:2:30:10:2:True LEADING:5 TRAILING:20 MINLEN:250 parameter. QC-passed reads were mapped to the assembled genome using Bowtie2^40^ with the no-mixed option (only proper pair read mapping). After final polishing, genome orientation and direction were identified using the ACT program v17.0.1^41^. Gene prediction and annotation of the constructed genome were conducted using Prokka v1.14.6^42^ and the closest neighbor of the assembled genome was identified using JSpeciesWS^43^. Completeness of the genome assembly was evaluated using BUSCO v5^44^ with streptomycetales_odb10. The GenBank accessions for wild-type *S. coelicolor* A3(2), mutant M22, and M22-2C43 were AL645882.2, CP133590, and CP133591, respectively.

### Comparative genome analysis

For genome map construction, Prokka v1.14.6 was used as the default parameter to obtain the predicted gene and annotation information for the wild type and two mutants. They maps were visualized using Proksee (https://proksee.ca/). To compare the variation information of the mutants with that of the wild-type strain, mapping was performed using the ACT program v18.2.0, and large genomic variations were obtained. Small genomic variations were obtained using GSalign v1.0.22^45^. As GSalign cannot identify the large structural variations, candidate regions identified in the ACT alignment were manually checked by mapping short reads. Additionally, snpEff v5.1^46^ was used to add annotation information for variations (parameters: -no-intron -no-intergenic -no-downstream -no-utr -upDownStreamLen 500). To identify closely related species possessing *dagB*, the *dagB* sequences of the mutants were annotated. However, the locus of *dagB* in the genome could not be specified, because Prokka uses the Swiss-Prot database, which contains a limited number of known proteins. Therefore, the gene sequence of SCO3487, known as *dagB* in wild-type, was obtained from NCBI and searched in the genome of mutants using blastn. The *dagB* sequence was searched in the NCBI Web blastn using the Refseq Genome Database, bacteria (taxid:2) option. After downloading 11 genomes of the species obtained from the search and extracting the *dagB* sequence, this sequence was compared with the *dagB* sequences of mutants M22 and M22-2C43. To determine changes in the amino acid sequence, the gene sequence was converted to a protein sequence for comparison. The predicted protein structure information of DagB was obtained using AlphaFold2 and the default parameter^47^, and protein pocket prediction programs (CASTp^48^ and DoGSiteScorer^49^). The structure was visualized using the Mol* 3D Viewer^50^ of the PDB (https://www.rcsb.org/) and compared using pairwise structure alignment.

### Molecular cloning and expression of DagB

DagB of the wild-type and DagB of mutant M22-2C43 were cloned into the pUWL201pw cloning vector; unprocessed pUWL201pw was used as a control. Thus, two *dagB* recombinants, wild-type and mutant M22-2C43, and a control pUWL201pw vector were used. Sco6545 (GenBank NP_630626) is an 890 amino acid with agarase enzyme properties of *S. coelicolor*. The 1,106-bp fragment (NdeI/BglII) encoding the N-terminal and the 1175-bp fragment (BglII/EcoRI) encoding the C-terminal of Sco6545 were ligated with pUWL201pw an NdeI-EcoRI restriction enzyme under the control of the emE* promoter to produce pUWL201-6545^22^. Each vector was transformed into the *Streptomyces lividans* TK24 strain, which lacks the β-agarase gene. The β-agarase specific activity of the supernatant was measured over for 3 days using the DNS method.

## Results and discussion

### β-agarase characteristic of wild-type *S. coelicolor* A3(2) and mutants M22 and M22-2C43

**Figure 1:**
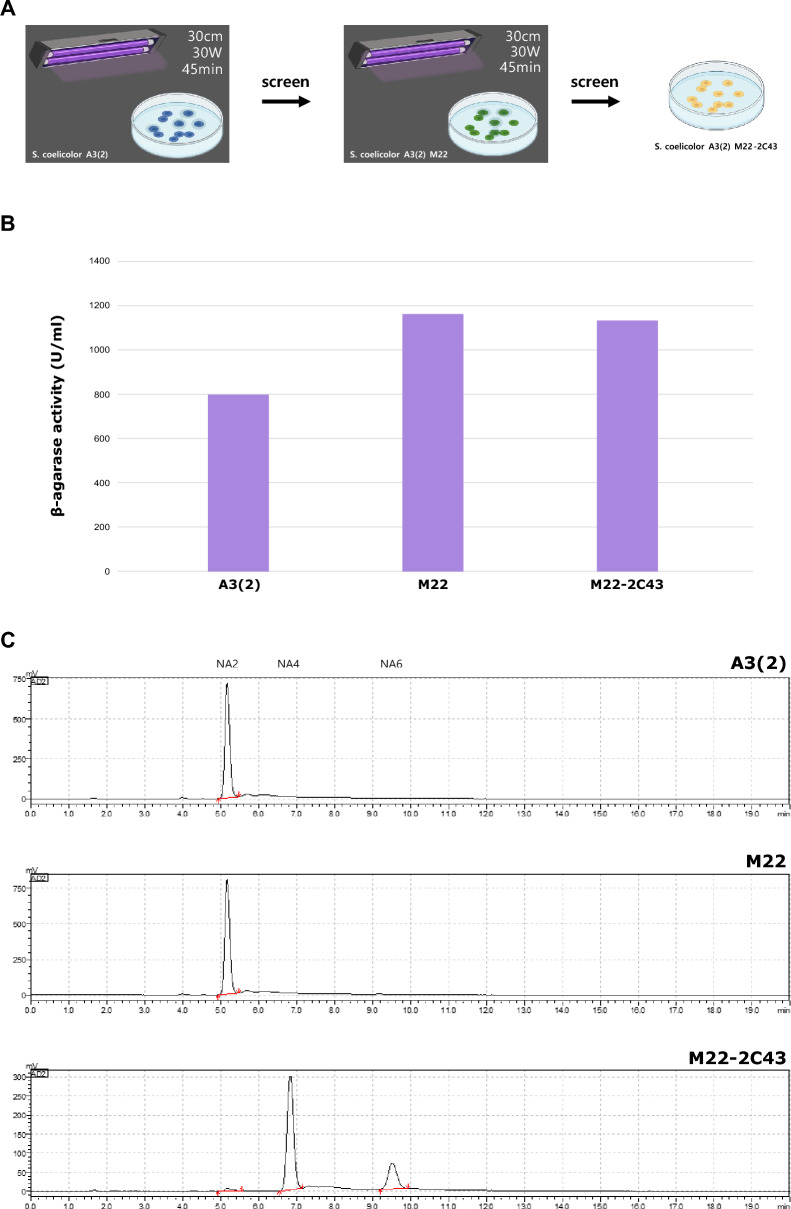
UV-induced mutagenesis and measurement of phenotypic characteristics. (**A**) Process of UV-induced mutagenesis from *S. coelicolor* A3(2) to *S. coelicolor* A3(2) M22-2C43. (**B**) The β-agarase activity of each strain. Activity was measured using the DNS method. The unit of β-agarase activity was U/mL. 1 U (Unit) of β-agarase activity was defined as activity with an absorbance of 0.001 at 540 nm. (**C**) Composition of neoagarooligosaccharide (NAO) in *S. coelicolor* A3(2), M22, and M22-2C43. NA2, neoagarobiose; NA4, neoagarotetraose; NA6, neoagarohexaose.

After UV-induced mutagenesis of the wild-type strain (John Innes Foundation, United Kingdom), the mutant was screened according to its clear zone size and spore-forming ability. Based on the DNS measurement, the mutant with the highest increase in β-agarase activity compared to the wild-type strain was screened and named *S. coelicolor* A3(2) M22. The mutant M22 cells were then treated with UV light and screened based on the criteria mentioned above. Colonies were classified based on their morphology, and the mutant with NA4/NA6 as the NAO composition in the HPLC-ELSD results was named *S. coelicolor* A3(2) M22-2C43. This process is briefly described in Fig. 1A. The size of the clear zone increased during mutagenesis in the agar medium, and was expected to increase the agar degradation ability. To verify this phenotype, the β-agarase activity of each strain was compared using the DNS method. Figure 1B shows the results of measuring β-agarase activity with a supernatant sample. Notably, the β-agarase activity of the mutant M22-2C43 was higher than that of wild-type. To identify the active β-agarase in each strain, the composition ratio of NAO was measured using HPLC-ELSD after the reaction with agarose. As mentioned previously, NA4/NA6 is produced by metabolizing agarose by DagA and NA2 is produced by metabolizing NA4/NA6 by DagB. As shown in Fig. 1C, the result of measuring NAO composition after reacting β-agarase of each strain with agarose revealed NA2 as the main product in the wild-type strain and mutant M22, and NA4/NA6 as the main product in mutant M22-2C43. Therefore, agarose degradation in mutant M22-2C43 was caused by DagA, which mainly produced NA4/NA6. This finding indicates that DagB, which degrades NA4/NA6 to NA2 during the agarose degradation process, was inactivated in mutant M22-2C43. Overall, these results indicate that mutant M22-2C43 has a higher β-agarase activity than the wild-type strain and produces NA4/NA6 as a final product from agarose using DagA.

### Changes in the complete genome during UV-induced mutagenesis

**Figure 2:**
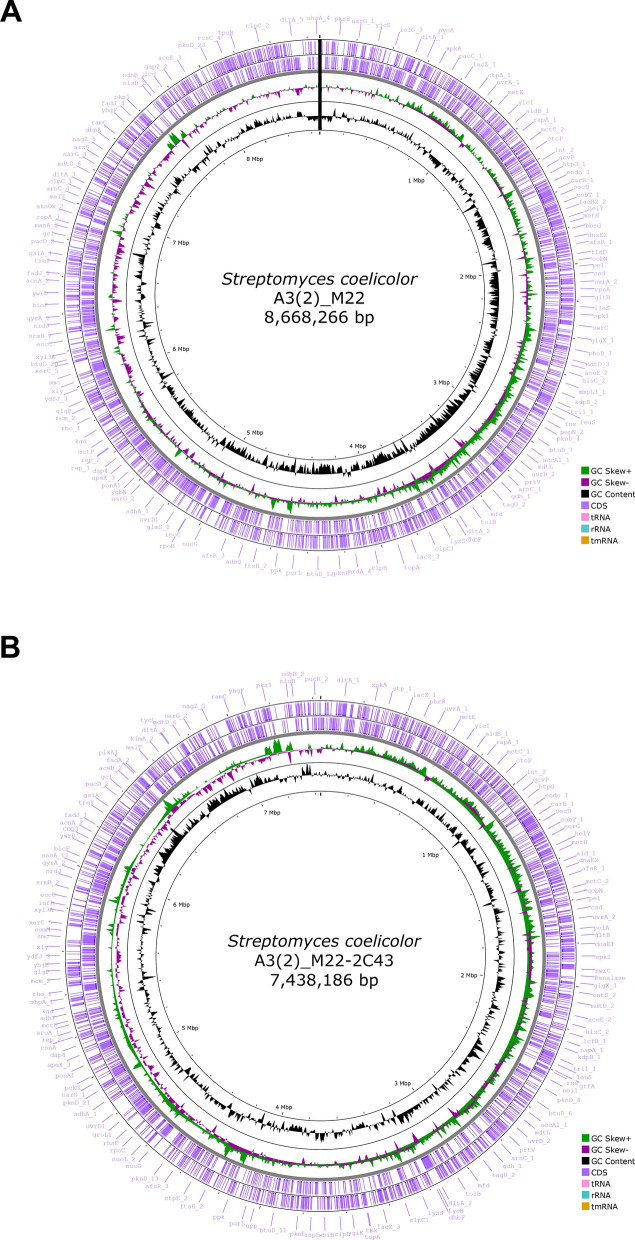
Circular chromosome map of the M22 and M22-2C43 strains. (**A**) Chromosome map of *S. coelicolor* A3(2) M22. Black line indicates a linear chromosome. (**B**) Chromosome map of *S. coelicolor* A3(2) M22-2C43.

To identify the characteristics of mutant M22-2C43 at the genetic level, comparative genome analysis was conducted. The complete genome sequences of the two mutants (M22 and M22-2C43) were successfully obtained using hybrid sequencing (Nanopore and Illumina); Table 1 shows the overview assembly statistics of the two assemblies with the wild type. To confirm the taxonomic species in the assembled genome, a tetracorrelation search was conducted using the JSpeciesWS. The results of the tetra correlation search revealed that *S. coelicolor* A3(2) was the closest genome to the two mutants (Z-Score 0.99993 and 099,975, respectively). The ANIb results also revealed that the two genomes had 100% ANIb values for *S. coelicolor* A3(2) (one direction). Two important features were identified due to the assembly of mutants M22 and M22-2C43. First, plasmids from both genomes were removed during mutagenesis. Second, the chromosome topology was changed from the linear chromosome of mutant M22 to the circular chromosome of mutant M22-2C43 through a second round of mutagenesis. The genetic instability of the *Streptomyces* linear chromosomes has already been studied, and both ends of the chromosome are known to be deleted by factors such as UV irradiation, causing the chromosome to change into a circular form^51,52^. In this study, a genome region of approximately 1.2 Mb was deleted at both ends of mutant M22-2C43, which is assumed to induce chromosome circularization. Figure 2 shows the circular chromosomal map of the two mutants. The GenBank accession number is CP133590 for *S. coelicolor* A3(2) M22 and CP133591 for *S. coelicolor* A3(2) M22-2C43.

**Table 1:** Whole genome sequence overview of the two strains with the reference genome.

Strain	A3(2)	M22	M22-2C43
Species name	*Streptomyces coelicolor*		
NCBI taxonomy ID	1902		
Domain	Bacteria		
Taxonomy	Bacteria; Terrabacteria group; Actinobacteria; Actinobacteria; Streptomycetales ; Streptomycetaceae ; Streptomyces; Streptomyces albidoflavus group; Streptomyces coelicolor		
Genome size (bp)	8,667,507	8,668,266	7,438,186
GC content in the DNA	72.12 mol% G + C	72.12 mol% G + C	72.15 mol% G + C
Number of genome Sequences	1 Linear (Single chromosomal DNA with 2 plasmids)	1 Linear (Single chromosomal DNA without plasmid)	1 Circular (Single chromosomal DNA without plasmid)
Number of plasmids	2	0	0
Number of coding sequences	7711	7711	6604
Number of rRNA (tRNA)	18(88)	18(88)	18(84)
Homology with *S. coelicolor* A3 (2) by JSpeciesWS (ANIb)	–	100%	100%

Mutant M22 was selected following a first round of UV-induced mutagenesis. To identify large genomic variations during mutagenesis, genome-to-genome alignment was conducted between wild-type and mutant M22 using ACT. Thirteen genomic variations were identified including four inversions, six insertions, and three deletions. Details of the variations are provided in Supplementary Table 1. To identify small genomic variations during mutagenesis, the genome sequence of mutant M22 was aligned with that of the wild-type using GSalign. GSalign revealed 154 genomic variations, including 29 insertions, 112 substitutions, and 13 deletions. Details of the variations are provided in Supplementary Table 2.

**Figure 3:**
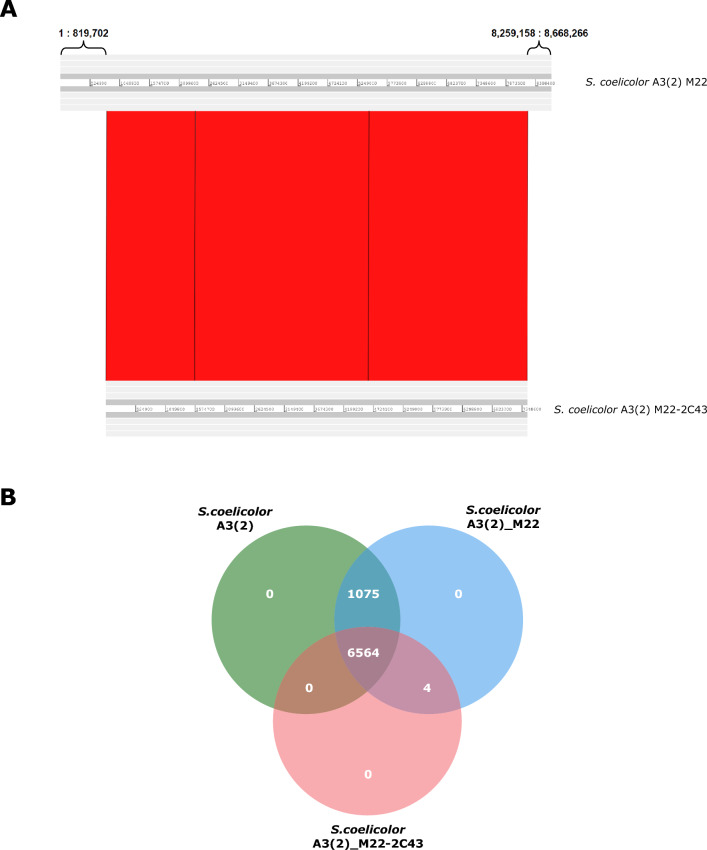
Large variations of the genome structure in M22-2C43 and Venn diagram of each gene. (**A**) Large deletions at both ends during the mutagenesis of *S. coelicolor* A3(2) M22 into *S. coelicolor* A3(2) M22-2C43. (**B**) Comparison of the number of predicted proteins between *S. coelicolor* A3(2), M22, and M22-2C43.

Mutant M22-2C43 was identified following a second round of UV-induced mutagenesis. Figure 3A shows large genomic variations through genome alignment between M22 and M22-2C43. Genome-to-genome alignment using ACT revealed that both ends of mutant M22 were deleted. After checking the three alignment blocks, additional large genomic variations were identified. This variation is summarized in Supplementary Table 3. For large insertion in mutant M22-2C43, the “CTCGGTG” motif repeatedly appeared as that found in the known wild-type genome. To identify small genomic variations during mutagenesis, the genome sequence of mutant M22-2C43 was aligned to that of M22 using GSalign. GSalign revealed 29 genomic variations, including two insertions, 22 substitutions, and five deletions. Details of the variations are provided in Supplementary Table 4.

To compare the specific gene content across the three strains and their functions, OrthoVenn2 was employed. Figure 3B shows the results of gene cluster comparison. The number of genes clustered in mutants M22 and M22-2C43 was 4 (obtained through mutagenesis from wild-type to M22), and the number of genes clustered in wild-type and mutant M22 was 1,075 (lost through mutagenesis from M22 to M22-2C43). Three of the four genes encoded hypothetical proteins, and the IS5 family transposase ISSgr2, was the only gene identified due to its function. No specific functions were identified based on the gene ontology terms of the unique clusters and genes of the two mutants. To identify the genes lost during mutagenesis, the gene list and Gene Ontology Biological Process were examined in relation to genes lost (1075) in mutant M22-2C43. Additional details are presented in Supplementary Table 5.

### Variations of the transport system in mutant M22

To verify the reason for the increased β-agarase activity in mutant M22 at the genetic level, 2000 bp up/downstream of *dagA* and *dagB*, which are predicted to contain regulatory sequences, were reviewed. Four promoter regions were identified for *dagA*^53^; however, no sequence changes were observed in either gene. In addition, *dagA* can be regulated by glucose repression. Therefore, the glucose kinase genes (*glk*)^54,55^ and SCO3485^56^, which play essential roles, were reviewed. However, no corresponding variations were observed. As a result, the variation genes were individually annotated, and the variation information was reviewed. The variation genes were annotated using snpEff v5.1 and gff information from *S. coelicolor* A3(2) (Refseq, GCF_000203835.1_ASM20383v1_genomic.gff). This information is presented in Supplementary Tables 1 and 2. The results of gene set enrichment analysis with FUNAGE-Pro^57^ for the variations were as follows: GO (2-oxoglutarate synthase activity, carbohydrate metabolic process, four iron, four sulfur cluster binding, tricarboxylic acid cycle, and respirasome) and KEGG (Glycolysis/Gluconeogenesis). Most of the variations in this table were annotated as non-coding regions or hypothetical proteins; however, some were associated with hypothetical membrane proteins and protein transport/export. *S. coelicolor* mainly uses the Tat system as a protein export pathway, and β-agarase is excreted through this pathway^58–60^. Although genes related to the Tat system were not detected in the list of variations, some genes were related to the ABC transport systems. Table 2 shows the variations, SCO5818 and SCO6512, which are related to the ABC transport system. The variation in SCO5818 were c.1367A > C and c.1368A > T, which resulted in a G456A mutation. The variation in SCO6512 was c.1468G > C, which resulted in a V490L mutation. Although variations that may be related to protein transport system or β-agarase activity were investigated as candidates through comparative genome analysis, an additional study is needed to identify the precise influence of these variations.

**Table 2:** List of genes related to protein transport among variations of M22.

Position	REF	ALT	Type of mutation	Gene	Description
6,365,315	A	C	MISSENSE	SCO5818	Putative ABC transporter
6,365,316	A	T	MISSENSE	SCO5818	Putative ABC transporter
7,203,532	G	C	MISSENSE	SCO6512	ABC transporter ATP-binding protein

### UV-induced mutagenesis causes loss of DagB activity in mutant M22-2C43

**Figure 4:**
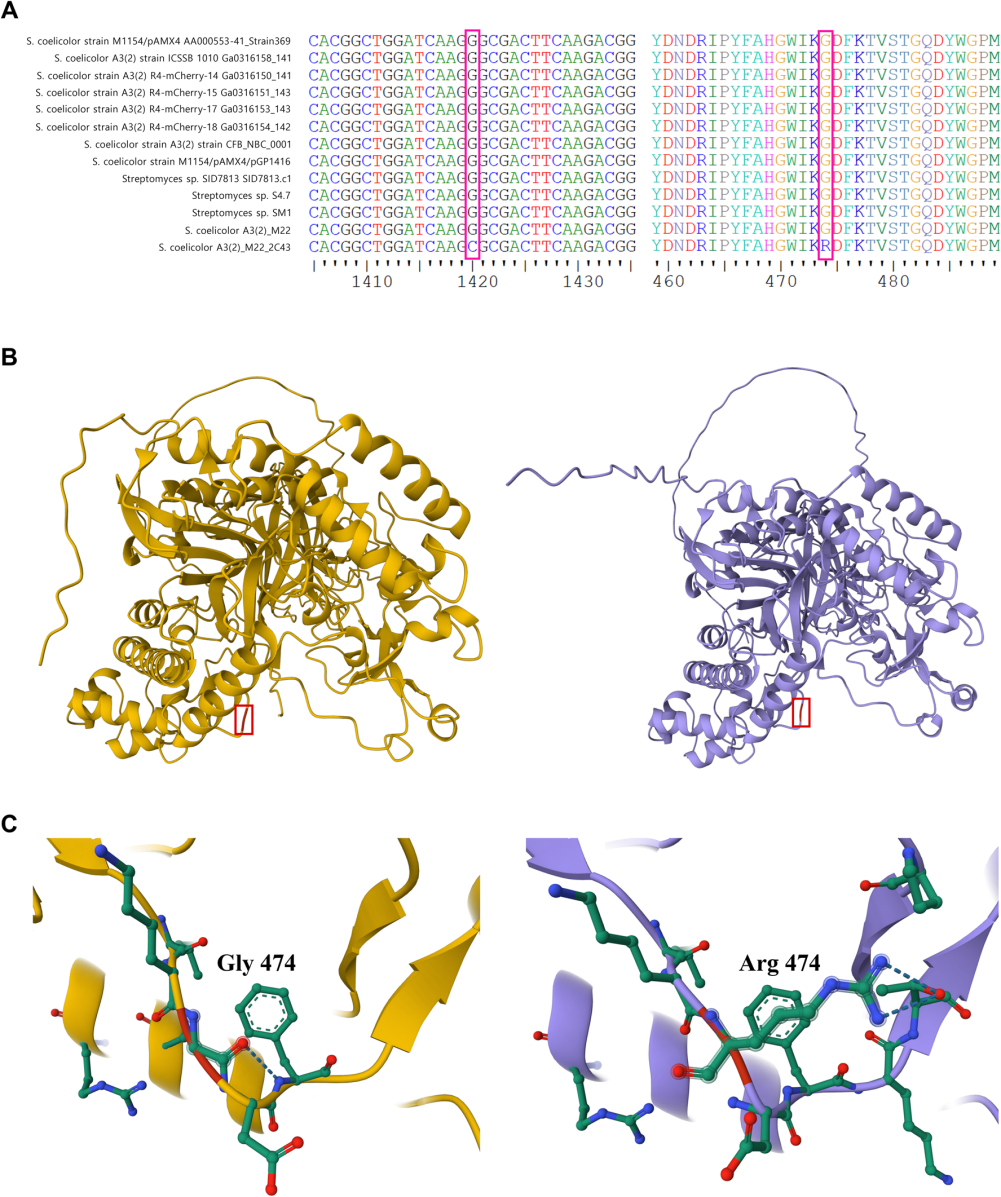
Comparison of the *dagB* genes, and modification of the DagB protein structures. (**A**) Comparison of the *dagB* sequences of closely related species. Gene sequences are shown on the left, amino acid sequences are shown on the right. (**B**) Prediction of the DagB protein structure using AlphaFold2. The boxed part represents mutated amino acids. (**C**) Mutation of the amino acid at position 474 and its surroundings (5 Å).

Based on the measured β-agarase activity and NAO composition (Fig. 1B,C), only DagA was found to function in agarose degradation in mutant M22-2C43. To compare this difference between strains at the molecular level, conservation of the *dagB* sequence (2397 bp) within closely related species was examined. The *dagB* gene of mutant M22 was located at 4,815,024:4,817,230 bp in the genome, which was a perfect match to the wild-type SCO3487 sequence. In contrast, *dagB* of mutant M22-2C43 was located at 3,993,669:3,996,065 bp in the genome and had one mismatch with the wild-type SCO3487 sequence. A comparison of the two sequences revealed that the *dagB* mutation in mutant M22-2C43 was c.1420G > C, resulting in G474A. To determine the level of *dagB* sequence conservation among closely related species, *dagB* was searched using blastn and was confirmed to be conserved in 11 strains. Figure 4A shows a comparison of the *dagB* sequences in 11 closely related species with the two mutants. The *dagB* gene was highly conserved, and one substitution mutation (c.1420G > C) was observed in mutant M22-2C43.

To identify the effect of amino acid sequence changes on the protein structure, AlphaFold2 was used to predict the DagB protein structures of the mutant and control. As shown in Fig. 4B, the structure of the protein changed, and the amino acid at position 474 and its predicted surroundings is presented in Fig. 4C. To measure the degree of protein structure change, a Pairwise Structure Alignment of PDB was used to compare the two protein structures. The following results were obtained: RMSD, 2.09; TM-Score, 0.95; Sequence Identity, 97%; Equivalent Residues, 779; Reference Coverage (M22 DagB), 98%; and Target Coverage (M22-2C43 DagB), 98%. After identifying the predicted protein structural changes, protein pocket prediction programs (CASTp and DoGSiteScorer) were used to determine whether G474A affected protein activity. Our pocket prediction analysis suggests that the G474A mutation has a substantial impact on the pocket structure of DagB. Supplementary Fig. 1 shows the predicted pockets of the DagB proteins. Among the amino acids, glycine (Gly) is the smallest with hydrogen atoms as its side chains. Gly is known to provide flexibility to the active site of proteins^61^. In contrast, arginine (Arg) is a relatively large amino acid with a positive charge at neutral pH. Therefore, Arg is a hydrophilic amino acid that tends to be mainly located on the surface of proteins^62^. In this study, the amino acid at position 474 in the predicted 3D structure of DagB was predicted to be located at the active site, and pocket prediction programs revealed that the amino acid at position 474 was located in the active site. In addition, by substituting the Gly at position 474 with Arg, which has a significantly different characteristics, the loss of DagB activity in mutant M22-2C43 was likely caused by G474A^63^.

**Figure 5:**
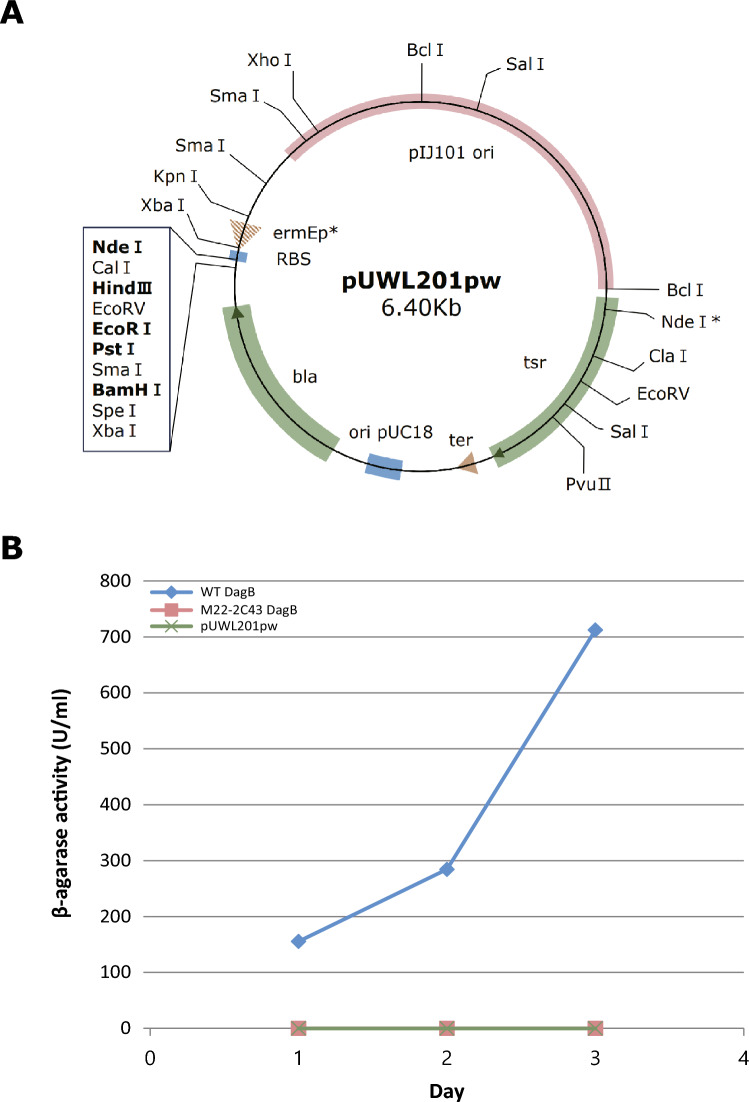
Activity of DagB β-agarase transformed into *Streptomyces lividans* TK24. (**A**) pUWL201pw vector was used in this study. (**B**) Activity measurements of wild-type, M22-2C43 DagB, and control (pUWL201pw) by date.

To prove the loss of function due to changes in the protein structure, recombinant vectors containing wild-type *dagB* and M22-2C43 *dagB* were transformed into *S. lividans* TK24 lacking the β-agarase gene, respectively. Figure 5A shows pUWL201pw used as the recombinant vector. By measuring the β-agarase activity, as expected from the structure prediction result, the activity was lost for M22-2C43 DagB. The results are shown in Fig. 5B. Thus, DagB function was confirmed to be lost due to a structural change caused by G474A without the involvement of other genes.

In this study, we generated *S. coelicolor* A3(2) M22-2C43, which produces NA4/NA6 without additional purification. The evolution of this mutant using UV-induced mutagenesis coupled with screening for increased β-agarase activity resulted in the loss of *dagB* due to a G474A mutation that likely alters its active site pocket. The catalytic activity of DagB was lost due to this structural change and affected the agarose degradation metabolism from NA4/NA6 to NA2. Consequently, DagA mainly acts on agarose degradation and produces NA4/NA6 as the final product in *S. coelicolor* A3(2) M22-2C43. Although some studies have reported the production of NA4/NA6 from *S. coelicolor* A3(2)^23,64^, this is the first study to use UV-induced mutagenesis. This study on mutant M22-2C43 producing NA4/NA6 exclusively is expected to offer an effective approach for large-scale production and a fundamental understanding of the application of this mutant. By utilizing this mutant, which selectively uses an enzyme or modifies the amino acid of DagB at position 474 using gene editing technologies, such as CRISPR, NA4/NA6 can be cost-effectively produced on a large scale. Furthermore, the understanding of such single-point mutation can contribute not only to *S. coelicolor* A3(2) but also to the enzymatic biotransformation of other strains known to express β-agarase.

## Conclusion

As the medical effect of NA4 and NA6 expands, our study focused on improving their efficient production. We used UV-induced mutagenesis to generate mutants M22 and M22-2C43 from the wild-type *S. coelicolor* A3(2) and found that β-agarase in mutant M22-2C43 produces the NA4/NA6 as a final product during agarose degradation. In addition, comparative genome analysis of β-agarase revealed that the DagB function was inactivated in mutant M22-2C43 due to a single nucleotide mutation in the conserved region, predicted as the DagB pocket site. In conclusion, mutant M22-2C43 efficiently produces NA4/NA6 as the final product from agarose, mainly through DagA activity, with increased β-agarase activity. This discovery could contribute to various applications, serving as a potential model for enzymatic biotransformation in strains expressing β-agarase.

### Supplementary Information

Supplementary Figure 1.

Supplementary Table 1.

Supplementary Table 2.

Supplementary Table 3.

Supplementary Table 4.

Supplementary Table 5.

Supplementary Legends.

## Data Availability

Sequence data and assembled complete genomes in this study have been deposited in the NCBI database with the accession number PRJNA987829. GenBank accession number is CP133590 for *S. coelicolor* A3(2) M22 and CP133591 for *S. coelicolor* A3(2) M22-2C43.
