# Supplementary material for: Construction of Streptomyces coelicolor A3(2) mutants that exclusively produce NA4/NA6 intermediates of agarose metabolism through mutation induction
Source: Sci Rep. 2023 Nov 3;13:18968. doi: 10.1038/s41598-023-46410-7 (PMC10624881; doi:10.1038/s41598-023-46410-7)
Supplement: Supplementary file 1 — Supplementary Figure 1. [file 41598_2023_46410_MOESM1_ESM.pdf]

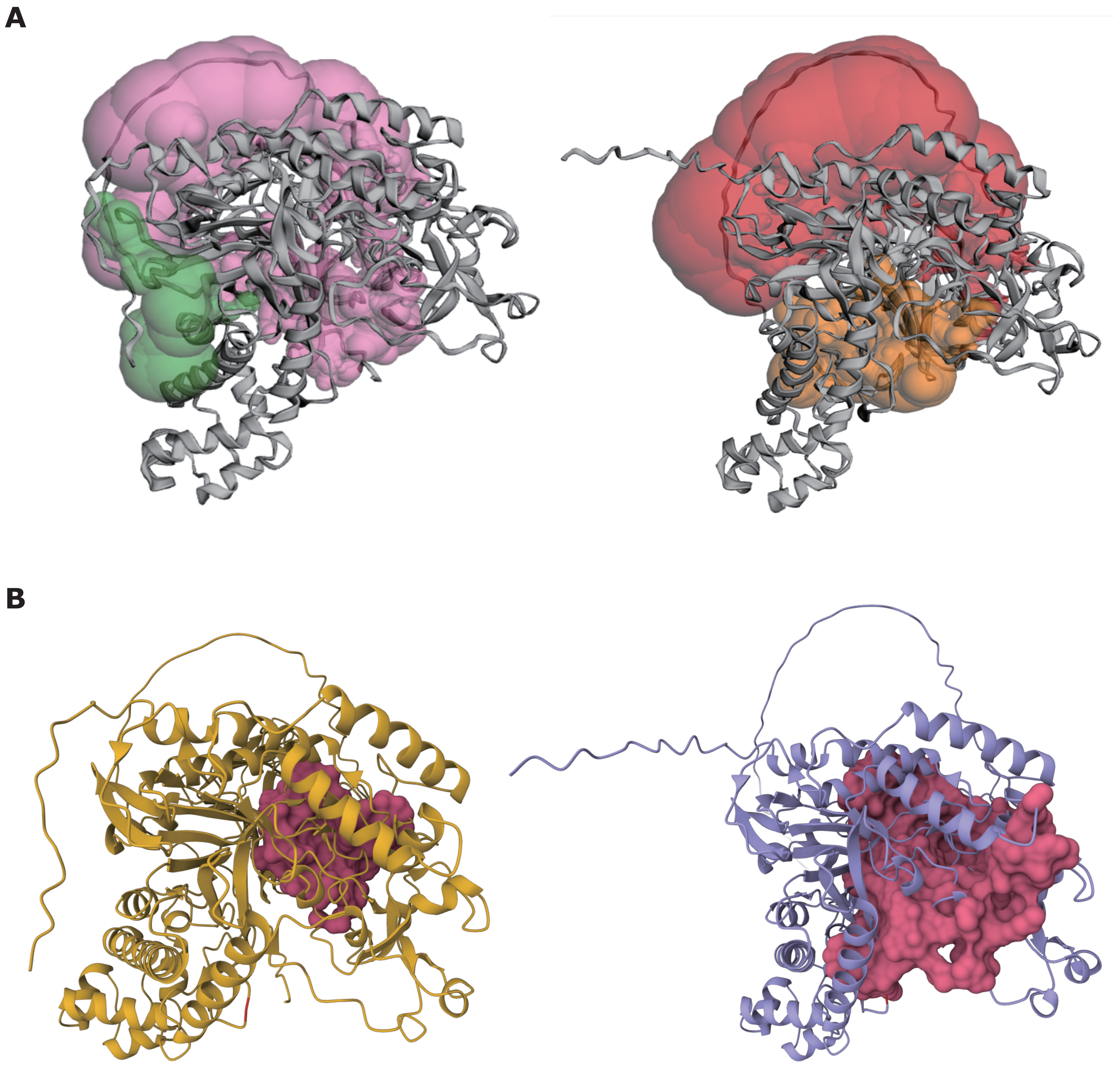

Supplementary Figure 1. Pocket structure of DagB predicted by CASTp and DoGSiteScorer.

(A) DagB pocket structure predicted with CASTp. Left: Structure of wild type DagB. The pink structure predicted as a pocket has the highest accuracy, and the green structure has the second highest accuracy. Right: Structure of M22-2C43 DagB. The red structure predicted as a pocket has the highest accuracy, and the orange structure has the second highest accuracy.

(B) DagB pocket structure predicted with DoGSiteScorer. Left: Structure of wild type DagB. Right: Struc ture of M22-2C43 DagB. Pink indicates the predicted pocket of the protein.
