# Supplementary material for: Construction of Streptomyces coelicolor A3(2) mutants that exclusively produce NA4/NA6 intermediates of agarose metabolism through mutation induction
Source: Sci Rep. 2023 Nov 3;13:18968. doi: 10.1038/s41598-023-46410-7 (PMC10624881; doi:10.1038/s41598-023-46410-7)
Supplement: Supplementary file 7 — Supplementary Legends. [file 41598_2023_46410_MOESM7_ESM.docx]

**Supplementary Figure 1.** Pocket structure of DagB predicted by CASTp and DoGSiteScorer.

1. DagB pocket structure predicted with CASTp. Left: Structure of wild type DagB. The pink structure predicted as a pocket has the highest accuracy, and the green structure has the second highest accuracy. Right: Structure of M22-2C43 DagB. The red structure predicted as a pocket has the highest accuracy, and the orange structure has the second highest accuracy.
2. DagB pocket structure predicted with DoGSiteScorer. Left: Structure of wild type DagB. Right: Structure of M22-2C43 DagB. Pink indicates the predicted pocket of the protein.

**Supplementary Table 1.** Large variations during UV-induced mutagenesis of *S. coelicolor* A3(2) to mutant M22.

**Supplementary Table 2.** Small variations during UV-induced mutagenesis of *S. coelicolor* A3(2) to mutant M22.

**Supplementary Table 3.** Large variations during UV-induced mutagenesis of mutant M22 to mutant M22-2C43.

**Supplementary Table 4.** Small variations during UV-induced mutagenesis of mutant M22 to mutant M22-2C43.

**Supplementary Table 5.** Specific clustered genes between *S. coelicolor* A3(2) and mutant M22 were predicted to the Cluster List and the term of Gene Ontology Biological Process using OrthoVenn2.
